# Increased blood flow prevents intramucosal acidosis in sheep endotoxemia: a controlled study

**DOI:** 10.1186/cc3021

**Published:** 2005-01-11

**Authors:** Arnaldo Dubin, Gastón Murias, Bernardo Maskin, Mario O Pozo, Juan P Sottile, Marcelo Barán, Vanina S Kanoore Edul, Héctor S Canales, Julio C Badie, Graciela Etcheverry, Elisa Estenssoro

**Affiliations:** 1 Medical Director, Intensive Care Unit, Sanatorio Otamendi y Miroli, Buenos Aires Argentina; 2 Staff Physician, Intensive Care Unit, Clinicas Bazterrica y Santa Isabel, Buenos Aires, Argentina; 3 Medical Director, Intensive Care Unit, Hospital Posadas, Buenos Aires, Argentina; 4 Research Fellow, Cátedra de Farmacología, Facultad de Ciencias Médicas, Universidad Nacional de La Plata, Argentina; 5 Medical Director, Renal Transplantation Unit, CRAI Sur, CUCAIBA, Argentina; 6 Staff Physician, Intensive Care Unit, Hospital San Martin de la Plata, Argentina; 7 Staff Physician, Clinical Chemistry Laboratory, Hospital San Martin de La Plata, Argentina; 8 Medical Director, Intensive Care Unit, Hospital San Martin de la Plata, Argentina

**Keywords:** Carbon dioxide, oxygen consumption, blood flow, endotoxemia, metabolic acidosis

## Abstract

**Introduction:**

Increased intramucosal–arterial carbon dioxide tension (PCO_2_) difference (ΔPCO_2_) is common in experimental endotoxemia. However, its meaning remains controversial because it has been ascribed to hypoperfusion of intestinal villi or to cytopathic hypoxia. Our hypothesis was that increased blood flow could prevent the increase in ΔPCO_2_.

**Methods:**

In 19 anesthetized and mechanically ventilated sheep, we measured cardiac output, superior mesenteric blood flow, lactate, gases, hemoglobin and oxygen saturations in arterial, mixed venous and mesenteric venous blood, and ileal intramucosal PCO_2 _by saline tonometry. Intestinal oxygen transport and consumption were calculated. After basal measurements, sheep were assigned to the following groups, for 120 min: (1) sham (*n *= 6), (2) normal blood flow (*n *= 7) and (3) increased blood flow (*n *= 6). *Escherichia coli *lipopolysaccharide (5 μg/kg) was injected in the last two groups. Saline solution was used to maintain blood flood at basal levels in the sham and normal blood flow groups, or to increase it to about 50% of basal in the increased blood flow group.

**Results:**

In the normal blood flow group, systemic and intestinal oxygen transport and consumption were preserved, but ΔPCO_2 _increased (basal versus 120 min endotoxemia, 7 ± 4 versus 19 ± 4 mmHg; *P *< 0.001) and metabolic acidosis with a high anion gap ensued (arterial pH 7.39 versus 7.35; anion gap 15 ± 3 versus 18 ± 2 mmol/l; *P *< 0.001 for both). Increased blood flow prevented the elevation in ΔPCO_2 _(5 ± 7 versus 9 ± 6 mmHg; *P *= not significant). However, anion-gap metabolic acidosis was deeper (7.42 versus 7.25; 16 ± 3 versus 22 ± 3 mmol/l; *P *< 0.001 for both).

**Conclusions:**

In this model of endotoxemia, intramucosal acidosis was corrected by increased blood flow and so might follow tissue hypoperfusion. In contrast, anion-gap metabolic acidosis was left uncorrected and even worsened with aggressive volume expansion. These results point to different mechanisms generating both alterations.

## Introduction

Rapid resolution of tissue hypoxia is the cornerstone of the treatment of sepsis and septic shock [1]. Patients who spontaneously develop high oxygen transport have better outcomes [2]. In experimental models of sepsis, animals with spontaneous elevation of oxygen transport present improved survival [3]. In addition, mortality from sepsis and septic shock could be reduced by early goal-directed therapy [4].

The intramucosal minus arterial carbon dioxide tension (PCO_2_) gradient (ΔPCO_2_) is considered a sensitive marker of regional gut perfusion [5] and is frequently found in human sepsis and in experimental endotoxemia. Because intramucosal acidosis can appear with normal or increased blood flow, it has been ascribed to a defect in cellular metabolism, namely cytopathic hypoxia [6]. It has also been related to decreased perfusion of villi [7]. Vasodilators might correct these microcirculatory deficits [8-10], but volume expansion or inotropic drugs have often failed to reverse intramucosal acidosis [11-14].

Our goal was to evaluate the effects of supranormal elevations of blood flow on oxygen transport and tissue oxygenation in a sheep model of endotoxemia. Our hypothesis was that increased blood flow could prevent the increase in ΔPCO_2 _and improve systemic metabolic acidosis.

## Methods

### Surgical preparation

Nineteen sheep were anesthetized with 30 mg/kg sodium pentobarbital, then intubated and mechanically ventilated (Dual Phase Control Respirator Pump Ventilator; Harvard Apparatus, South Natick, MA, USA) with a tidal volume of 15 ml/kg, a fraction of inspired oxygen (F_I_O_2_) of 0.21 and positive end-expiratory pressure adjusted to maintain O_2 _arterial saturation at more than 90%. The respiratory rate was set to keep the end-tidal PCO_2 _at 35 mmHg. Neuromuscular blockade was performed with intravenous pancuronium bromide (0.06 mg/kg). Additional pentobarbital boluses (1 mg/kg per hour) were administered as required.

Catheters were advanced through the left femoral vein to administer fluids and drugs, and through the left femoral artery to measure blood pressure and to obtain blood gases. A pulmonary artery catheter was inserted through right external jugular vein (Flow-directed thermodilution fiberoptic pulmonary artery catheter; Abbott Critical Care Systems, Mountain View, CA, USA).

An orogastric tube was inserted to allow drainage of gastric contents. A midline laparotomy and splenectomy were then performed. An electromagnetic flow probe was placed around the superior mesenteric artery to measure intestinal blood flow. A catheter was placed in the mesenteric vein through a small vein proximal to the gut to draw blood gases. A tonometer was inserted through a small ileotomy to measure intramucosal PCO_2_. Lastly, after careful hemostasis, the abdominal wall incision was closed.

### Measurements and derived calculations

Arterial, systemic, pulmonary and central venous pressures were measured with corresponding transducers (Statham P23 AA; Statham, Halo Rey, Puerto Rico). Cardiac output was measured by thermodilution with 5 ml of saline solution (HP OmniCare Model 24 A 10; Hewlett Packard, Andover, MA, USA) at 0°C. An average of three measurements taken randomly during the respiratory cycle were considered and were normalized to body weight to yield Q. Intestinal blood flow was measured by the electromagnetic method (Spectramed Blood Flowmeter model SP 2202 B; Spectramed Inc., Oxnard, CA, USA) with *in vitro *calibrated transducers 5–7 mm in diameter (Blood Flowmeter Transducer; Spectramed Inc.). Occlusive zero was controlled before and after each experiment. Non-occlusive zero was corrected before each measurement. Superior mesenteric blood flow was normalized to gut weight (Q_intestinal_).

Arterial, mixed venous and mesenteric venous partial pressure of oxygen (PO_2_), PCO_2 _and pH were measured with a blood gas analyzer (ABL 5; Radiometer, Copenhagen, Denmark), and hemoglobin and oxygen saturation were measured with a co-oximeter calibrated for sheep blood (OSM 3; Radiometer). Arterial, mixed venous and mesenteric venous contents (C_a_O_2_, C_v_O_2 _and C_vm_O_2_, respectively) were calculated as (Hb × 1.34 × O_2 _saturation) + (PO_2 _× 0.0031). Systemic and intestinal oxygen transport and oxygen consumption (DO_2_, VO_2_, DO_2i _and VO_2i_, respectively) were calculated as DO_2 _= Q × C_a_O_2_; VO_2 _= Q × (C_a_O_2 _- C_v_O_2_); DO_2i _= Q_intestinal _× C_a_O_2_, and VO_2i _= Q_intestinal _× (C_a_O_2 _- C_vm_O_2_).

Intramucosal PCO_2 _was measured with a tonometer [15] (TRIP Sigmoid Catheter; Tonometrics, Inc., Worcester, MA, USA) filled with 2.5 ml of saline solution; 1.0 ml was discarded after an equilibration period of 30 min and PCO_2 _was measured in the remaining 1.5 ml. Its value was corrected to the corresponding equilibration period and was used to calculate ΔPCO_2_.

Mixed venous–arterial and mesenteric venous–arterial PCO_2 _differences were also calculated. Arterial, mixed venous and mesenteric venous CO_2 _contents (CCO_2_) and their differences were calculated with Douglas's algorithm [16]. Systemic and intestinal CO_2 _production (VCO_2 _and VCO_2i_, respectively) were calculated as VCO_2 _= Q × mixed venoarterial CCO_2_, and VCO_2i _= Q_intestinal _× mesenteric venoarterial CCO_2_. Global blood capacity for transporting CO_2 _was evaluated as the ratio between venoarterial CCO_2 _and PCO_2 _differences (R_a-v_). This index has been used to evaluate the amount of CO_2 _transported by the blood in relation to the venoarterial gradient of PCO_2 _[17].

Lactate, sodium, potassium, chloride and serum total proteins were measured with an automatic analyzer every 60 min (Automatic Analyzer Hitachi 912; Boehringer Mannheim Corporation, Indianapolis, IN, USA). Anion gap was calculated as ([Na^+^] + [K^+^]) - ([Cl^-^] + [HCO_3_^-^]). Anion gap was corrected for changes in plasma protein concentration [18].

### Experimental procedure

Basal measurements were taken after a stabilization period longer than 30 min. Then animals were assigned to the following groups: (1) sham group (*n *= 6), consisting of sheep receiving 100 ml of saline in 10 min, followed by an infusion necessary to keep intestinal blood flow at basal levels; (2) normal blood flow group (*n *= 7), consisting of sheep receiving 5 μg/kg *Escherichia coli *lipopolysaccharide dissolved in 100 ml of saline in 10 min, and then saline infusion so as to maintain intestinal blood flow at basal levels; and (3) increased blood flow group (*n *= 6), consisting of sheep receiving 5 μg/kg *Escherichia coli *lipopolysaccharide dissolved in 100 ml of saline in 10 min, followed by saline infusion so as to increase intestinal blood flow by 50% from basal levels.

F_I_O_2 _was increased to 0.50 in endotoxemic sheep to avoid deep hypoxemia.

Measurements were performed at 30 min intervals for 120 min from the start of endotoxin administration.

At the end of the experiment, the animals were killed with an additional dose of pentobarbital and a KCl bolus. A catheter was inserted in the superior mesenteric artery and Indian ink was instilled through it. Dyed intestinal segments were dissected, washed and weighed for the calculation of gut indexes.

The local Animal Care Committee approved the study. Care of animals was in accordance with National Institute of Health guidelines.

### Statistical analysis

Data were assessed for normality and expressed as means ± SD. Differences within groups were analyzed with a repeated-measures analysis of variance and Dunnett's multiple comparisons test to compare each time point with basal. One-time comparisons between groups were tested with a one-way analysis of variance and a Newman–Keuls multiple comparison test.

## Results

### Hemodynamic and oxygen transport effects

Sham, normal blood flow and increased blood flow groups received 10 ± 6, 24 ± 9 and 91 ± 38 ml/kg per hour, respectively, of normal saline solution (*P *< 0.05) to achieve resuscitation goals. Variations of intestinal blood flow from basal values, at the end of the experiment, were 8 ± 5%, – 1 ± 22% and 60 ± 22%, respectively (*P *< 0.05). As expected, the increased blood flow group had higher central venous and pulmonary wedge pressures, intestinal blood flow, cardiac output and systemic oxygen transport than the normal blood flow group. The increased blood flow group had also higher intestinal oxygen consumption (Table 1).

### Metabolic effects

**Figure 1:**
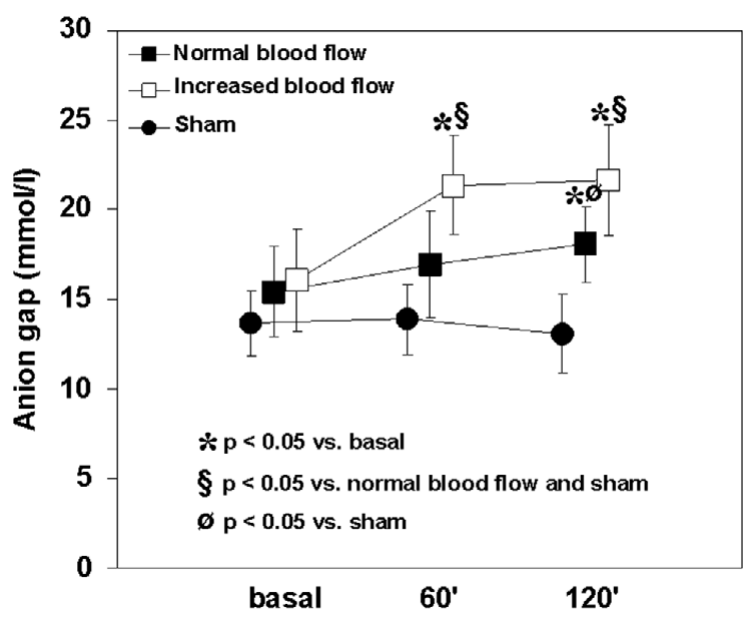
Behavior of the anion gap in the sham, normal and increased blood flow groups. A higher degree of anion-gap metabolic acidosis developed in the increased blood flow group than in the normal blood flow group. The anion gap was unchanged in the sham group. 60' and 120' refer to 60 and 120 min, respectively.

Metabolic acidosis developed in both groups with endotoxemia, but was greater in the increased blood flow group because of hyperchloremia and an increased anion gap (Table 2 and Fig. 1). These variables did not change in the sham group. Lactate levels remained stable in the three groups (Table 2).

### Effects on ΔPCO2 and its determinants

**Figure 2:**
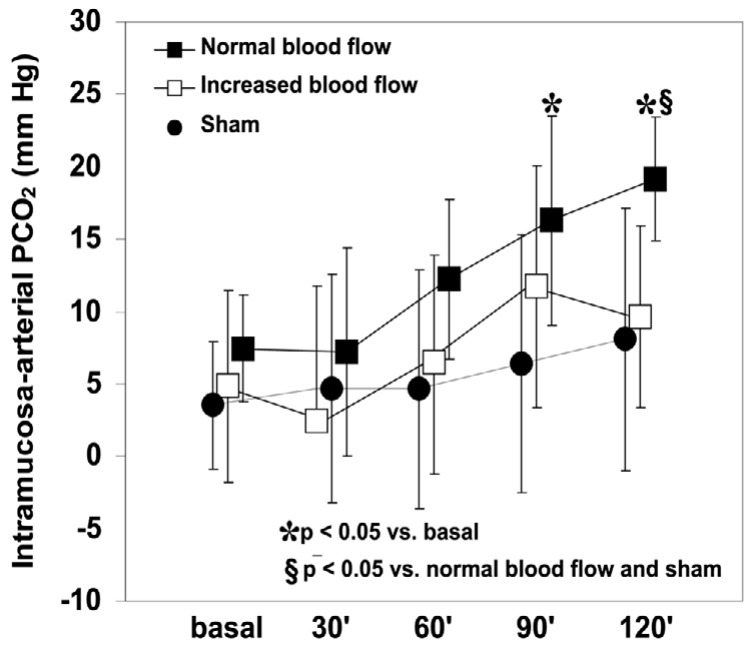
Behavior of intramucosal – arterial PCO_2 _difference in the sham, normal and increased blood flow groups. Intramucosal acidosis developed in the normal blood flow group and was prevented in the increased blood flow group. Intramucosal – arterial PCO_2 _difference was unchanged in the sham group. 30', 60', 90' and 120' refer to 30, 60, 90 and 120 min, respectively.

ΔPCO_2 _increased in the normal blood flow group and remained unchanged in the increased blood flow and sham groups (Fig. 2). Systemic and intestinal venoarterial PCO_2 _differences were also higher in the normal blood flow group than in the others (Table 3). Systemic and intestinal R_a-v _were lower in both endotoxemic groups.

## Discussion

The main finding of this study was that increased blood flow prevented the development of intramucosal acidosis. However, anion-gap metabolic acidosis was larger in hyperresuscitated animals. These results underscore the different underlying mechanisms of each type of acidosis.

### The experimental model of endotoxemia

We used a short-term infusion of endotoxin followed by saline expansion to induce a state of normodynamic shock, with preserved cardiac output and intestinal blood flow [19,20]. A state of normodynamic shock was chosen as a control group to avoid CO_2 _accumulation caused by macrovascular hypoperfusion. We found that intramucosal acidosis and systemic metabolic acidosis occurred, in spite of stable systemic and gut oxygen transports and consumptions.

The reason for increased intestinal ΔPCO_2 _in sepsis remains controversial [21]. It might reflect hypoperfusion, but has also been found in normodynamic states [22]. Vallet and colleagues studied endotoxemic dogs with low blood flow, resuscitated with dextran. Gut flow was increased and oxygen transport normalized, but oxygen uptake and mucosal PO_2 _and pH remained low, results that were ascribed to flow redistribution from mucosal to serosal layers [13]. Conversely, Revelly and colleagues described flow redistribution from serosa to mucosa induced by endotoxin [23]. VanderMeer and colleagues found that intramucosal acidosis developed despite preserved blood flow and tissue PO_2 _in endotoxemic pigs, attributed to changes in energetic metabolism [24]. Thus, the concept of 'cytopathic hypoxia' was introduced [6].

However, cytopathic hypoxia and increased anaerobic CO_2 _production might not be the sole explanation for the increase in ΔPCO_2_. Vallet and colleagues [25] and Dubin and colleagues [26] recently showed that hypoperfusion is a key factor in the development of venous and tissue hypercarbia. In addition, Tugtekin and colleagues showed an association between increased ΔPCO_2 _and diminished villi microcirculation [7].

This body of information suggests that intramucosal acidosis in sepsis is due mainly to microcirculatory alterations, even though cardiac output and regional flows might remain unchanged. Disturbed energetic metabolism might be present in sepsis, but it does not explain intramucosal acidosis. However, it might be a reasonable explanation for the development of systemic metabolic acidosis in our experiments. Increased anion-gap metabolic acidosis appeared despite preserved oxygen metabolism. As described previously, metabolic acidosis was not explained by elevations of lactate but by increases in unmeasured anions whose source and identification are still unknown [27,28].

### Effects of saline solution expansion on intramucosal acidosis

Increased blood flow by volume expansion prevented ΔPCO_2 _elevation. PCO_2 _gradients, venoarterial and tissue-arterial PCO_2 _differences are the result of interactions between CO_2 _production, blood capacity to transport CO_2 _and blood flow to tissues. We and others have previously shown that ΔPCO_2 _fails to reflect tissue hypoxia when blood flow is preserved [25,26,29]. Our results suggest that intramucosal acidosis is related mainly to local hypoperfusion, because the only difference between our groups, in terms of PCO_2 _difference determinants, was the level of blood flow. We can speculate that volume expansion might improve microcirculation and, subsequently, CO_2 _clearance. However, intramucosal acidosis might be corrected by the inhibition of inducible nitric oxide synthase and without microcirculatory recruitment [30]. Improvement of cellular metabolism and/or redistribution of blood flow from the mucosa to other layers have been proposed as underlying mechanisms. We cannot exclude the possibility that increases in blood flow might decrease tissue hypoxia and anaerobically generated CO_2_. Intestinal VO_2 _increased after elevation of O_2 _transport in the increased blood flow group, suggesting unmet needs in the normal blood flow group. Flow might have been inadequate in the face of increased metabolic requirements caused by endotoxemia [31].

Despite this apparent dependence on intestinal oxygen supply, CO_2 _production remained stable. Possible reasons are error propagation in the VO_2 _and VCO_2 _calculations, or an increase in VO_2 _due to non-metabolic processes, such as the production of inflammatory reactants and reactive oxygen species [32].

Other investigators have reported that volume expansion could not correct intramucosal acidosis, in both clinical and experimental settings [11,13,14]. Differences in the level of attained blood flow, timing of expansion or the type of injury might account for these findings opposite to ours.

Potential limitations of our study are related to the errors of saline tonometry, such as inadequate equilibration time, deadspace effect and underestimation of PCO_2 _by blood gas analyzers [33,34].

### Effects of saline solution expansion on metabolic acidosis

Metabolic acidosis was a prominent finding in our study. Expansion with large volumes of saline predictably produced hyperchloremic metabolic acidosis [35]. In addition, metabolic acidosis arose as a result of unmeasured anions. Previous research has shown that during streptococcal infusion in pigs, metabolic acidosis decreased, but did not disappear, when oxygen transport was supported with dextran and red blood cells [36].

The reason for augmented unmeasured anions in the increased blood flow group is unclear. Possible causes are washout of tissue acids by high blood flow, or an impairment of oxygenation caused by tissue edema. Nevertheless, Gow and colleagues have shown that oxygen extraction is already altered in septic animals, so increased diffusion distances would not be relevant [37].

In addition, hyperchloremic acidosis might induce an inflammatory response, cellular dysfunction and apoptosis, and increased mortality in experimental septic shock [38-41]. In this way, a deleterious effect of acidosis on cellular function with the subsequent production of unknown anions might be operative.

## Conclusions

Despite preserved blood flow and oxygen transport, intramucosal acidosis developed in endotoxemic sheep. Volume expansion prevented the increase in ΔPCO_2_, implying that intramucosal acidosis is related mainly to local hypoperfusion. Despite aggressive expansion, anion-gap metabolic acidosis worsened, which suggests an effect on cellular metabolism.

## Key messages

• 	Intramucosal acidosis developed in endotoxemic sheep, despite preserved blood flow and oxygen transport.

• 	Increased blood flow prevented elevation in ΔPCO_2_, suggesting that intramucosal acidosis is mainly related to local hypoperfusion. However, anion-gap metabolic acidosis was higher, pointing to a possible effect on cellular mechanism.

## Abbreviations

C_a_O_2 _= arterial oxygen content; CCO_2 _= CO_2 _content; C_vm_O_2 _= mesenteric venous oxygen content; C_v_O_2 _= mixed venous oxygen content; DO_2 _= systemic oxygen transport; DO_2i _= intestinal oxygen transport; ΔPCO_2 _= intramucosal minus arterial PCO_2 _gradient; F_I_O_2 _= fraction of inspired oxygen; PCO_2 _= carbon dioxide tension; PO_2 _= partial pressure of oxygen; Q = cardiac output; Q_intestinal _= intestinal blood flow; R_a-v _= global blood capacity for transporting CO_2_; VCO_2 _= systemic CO_2 _production; VCO_2i _= intestinal CO_2 _production; VO_2 _= systemic oxygen consumption; VO_2i _= intestinal oxygen consumption.

## Competing interests

The author(s) declare that they have no competing interests.

## Authors' contributions

AD was responsible for the study concept and design, the analysis and interpretation of data, and drafting of the manuscript. GM, MOP, VSKE and HSC performed the acquisition of data and contributed to the draft of the manuscript. BM and GE conducted the blood determinations and contributed to the draft of the manuscript. MB and JPS performed the surgical preparation and contributed to the discussion. EE helped in the draft of the manuscript and made a critical revision for important intellectual content. All authors read and approved the final manuscript.

## Figures and Tables

**Table 1:** Systemic and intestinal hemodynamic and oxygen transport parameters in sham, normal and increased blood flow groups

Parameter	Group	Basal	Endotoxemia			
			
			30 min	60 min	90 min	120 min
Mean arterial pressure (mmHg)	Sham	81 ± 10	85 ± 15	88 ± 15	91 ± 16	92 ± 19
	Normal	93 ± 19	89 ± 25	83 ± 23	91 ± 32	94 ± 26
	Increased	90 ± 17	98 ± 17	89 ± 18	89 ± 21	99 ± 17
Mean pulmonary arterial pressure (mmHg)	Sham	16 ± 3	15 ± 3	16 ± 3	15 ± 4	16 ± 4
	Normal	15 ± 5	34 ± 9*†	26 ± 8*†	25 ± 7*†	24 ± 6*†
	Increased	20 ± 4	35 ± 10*†	31 ± 4*†	34 ± 6*†‡	35 ± 6*†‡
Pulmonary wedge pressure (mmHg)	Sham	5 ± 2	5 ± 2	5 ± 1	5 ± 2	5 ± 2
	Normal	5 ± 2	11 ± 4*†	8 ± 2*†	8 ± 3*†	8 ± 4
	Increased	6 ± 1	11 ± 4*†	13 ± 6*†	12 ± 3*†	14 ± 5*†‡
Central venous pressure (mmHg)	Sham	5 ± 5	5 ± 3	6 ± 5	5 ± 4	5 ± 4
	Normal	4 ± 2	5 ± 3	6 ± 2	6 ± 2	5 ± 3
	Increased	4 ± 2	8 ± 3	9 ± 5*	10 ± 4*†‡	11 ± 4*†‡
Cardiac output (ml/kg per min)	Sham	134 ± 30	148 ± 36	153 ± 37	144 ± 33	151 ± 41
	Normal	139 ± 43	117 ± 27	135 ± 38	149 ± 42	142 ± 34
	Increased	157 ± 51	221 ± 64*†‡	257 ± 67*†‡	276 ± 84*†‡	290 ± 91*†‡
Superior mesenteric artery blood flow (ml/min per g)	Sham	498 ± 107	568 ± 126*	551 ± 126*	548 ± 134*	539 ± 131*
	Normal	553 ± 184	514 ± 152	566 ± 161	573 ± 145	529 ± 169
	Increased	578 ± 206	803 ± 226*‡	794 ± 209*†‡	863 ± 326*‡	923 ± 370*†‡
	Increased	362 ± 116	437 ± 75†‡	286 ± 53	336 ± 102	295 ± 75
Systemic oxygen transport (ml/min per kg)	Sham	16.2 ± 4.5	18.0 ± 5.6*	19.0 ± 6.2*	17.8 ± 5.3	18.8 ± 6.1*
	Normal	16.4 ± 6.6	13.3 ± 4.9	14.0 ± 4.8	16.4 ± 6.4	15.8 ± 5.7
	Increased	17.2 ± 4.0	23.0 ± 5.5*‡	25.5 ± 6.7*‡	26.0 ± 8.4*‡	26.9 ± 9.9*‡
Systemic oxygen consumption (ml/min per kg)	Sham	6.4 ± 0.8	6.4 ± 1.1	6.8 ± 1.3	6.6 ± 1.2	7.2 ± 1.3
	Normal	6.4 ± 1.2	5.3 ± 1.2*	5.8 ± 1.6*	6.0 ± 1.5	6.5 ± 1.4
	Increased	7.6 ± 0.9	7.6 ± 2.0‡	7.3 ± 2.1	7.4 ± 2.2	8.3 ± 3.2
Intestinal oxygen transport (ml/min per kg)	Sham	62.3 ± 22.2	71.4 ± 24.8*	70.8 ± 25.1*	69.9 ± 24.6*	69.1 ± 24.0*
	Normal	64.0 ± 22.6	56.1 ± 19.3	57.0 ± 15.8	60.8 ± 18.4	56.5 ± 17.0
	Increased	64.3 ± 16.7	86.4 ± 19.1*‡	81.4 ± 22.1*	82.2 ± 23.5*	87.1 ± 23.6*‡
Intestinal oxygen consumption (ml/min per kg)	Sham	21.7 ± 4.0	21.1 ± 3.7	22.0 ± 3.2	22.7 ± 4.2	21.8 ± 4.7
	Normal	21.2 ± 4.1	22.1 ± 6.5	22.7 ± 8.9	22.6 ± 7.8	22.4 ± 9.0
	Increased	29.3 ± 9.7	28.9 ± 9.3	32.5 ± 13.0	29.8 ± 9.4	37.2 ± 12.3†‡

* *P *< 0.05 versus basal. † *P *< 0.05 versus sham. ‡ *P *< 0.05 versus normal. Sham, sham group; normal, normal blood flow group; increased, increased blood flow group.

**Table 2:** Arterial hemoglobin, acid-base and metabolic parameters in sham, normal and increased blood flow groups

Parameter	Group	Basal	Endotoxemia			
			
			30 min	60 min	90 min	120 min
Hemoglobin (g/l)	Sham	9.6 ± 2.4	9.7 ± 2.7	9.9 ± 2.3	9.8 ± 2.2	9.9 ± 2.2
	Normal	9.1 ± 2.3	9.0 ± 2.4	8.4 ± 2.0*	8.1 ± 2.2*	8.3 ± 2.4*
	Increased	8.9 ± 2.2	8.2 ± 2.3*	7.8 ± 2.4*	7.6 ± 2.5*	7.7 ± 2.5*
pH	Sham	7.44 ± 0.03	7.45 ± 0.02	7.45 ± 0.03	7.47 ± 0.02	7.47 ± 0.03
	Normal	7.39 ± 0.07	7.34 ± 0.08*†	7.31 ± 0.05*†	7.34 ± 0.05*†	7.35 ± 0.06*†
	Increased	7.42 ± 0.04	7.35 ± 0.05*†	7.31 ± 0.05*†	7.28 ± 0.08*†	7.25 ± 0.08*†‡
PCO_2 _(mmHg)	Sham	35 ± 3	34 ± 3	34 ± 3	33 ± 3	34 ± 4
	Normal	35 ± 4	38 ± 6*	41 ± 7*	37 ± 6	35 ± 6
	Increased	34 ± 2	36 ± 5	34 ± 3	34 ± 5	37 ± 6
PO_2 _(mmHg)	Sham	85 ± 13	88 ± 18	86 ± 16	88 ± 17	84 ± 15
	Normal	87 ± 16	119 ± 59	105 ± 39	123 ± 20*†	134 ± 43*†
	Increased	90 ± 23	150 ± 48*†	132 ± 21*†	101 ± 20	99 ± 31
[HCO_3_^-^] (mmol/l)	Sham	24 ± 2	24 ± 3	24 ± 3	24 ± 3	24 ± 3
	Normal	21 ± 2	21 ± 2	20 ± 2†	20 ± 2*†	19 ± 2*†
	Increased	22 ± 3	20 ± 2*†	17 ± 3*†	16 ± 3*†‡	16 ± 2*†‡
Base excess (mmol/l)	Sham	1 ± 3	1 ± 3	1 ± 3	2 ± 3	2 ± 3
	Normal	t2 ± 4	t5 ± 3*†	t5 ± 2*†	t5 ± 3*†	t5 ± 3*†
	Increased	t1 ± 4	t4 ± 3*†	t8 ± 4*†	t10 ± 4*†	t10 ± 3*†‡
[Cl^-^]/[Na^+^]	Sham	0.76 ± 0.02		0.76 ± 0.03		0.76 ± 0.03
	Normal	0.76 ± 0.01		0.77 ± 0.02		0.77 ± 0.01
	Increased	0.76 ± 0.02		0.78 ± 0.02*		0.80 ± 0.02*†‡
Lactate (mmol/l)	Sham	2.1 ± 0.7		2.0 ± 0.7		1.8 ± 0.6
	Normal	1.7 ± 0.8		1.9 ± 0.7		2.2 ± 1.1
	Increased	2.2 ± 1.6		1.7 ± 1.1		1.9 ± 1.1

* *P *< 0.05 versus basal. † *P *< 0.05 versus sham. ‡ *P *< 0.05 versus normal. Sham, sham group; normal, normal blood flow group; increased, increased blood flow group.

**Table 3:** Systemic and intestinal CO_2_-derived parameters in sham, normal and increased blood flow groups

Parameter	Group	Basal	Endotoxemia			
			
			30 min	60 min	90 min	120 min
Mixed venous – arterial PCO_2 _(mmHg)	Sham	6 ± 2	6 ± 2	6 ± 2	6 ± 2	5 ± 2
	Normal	7 ± 2	8 ± 2	7 ± 2	8 ± 3	8 ± 3†
	Increased	6 ± 2	6 ± 3	7 ± 5	7 ± 4	4 ± 1‡
Mesenteric venous – arterial PCO_2 _(mmHg)	Sham	6 ± 2	5 ± 2	5 ± 2	6 ± 2	5 ± 2
	Normal	7 ± 2	8 ± 2	8 ± 3	10 ± 4	10 ± 2*†
	Increased	8 ± 3	6 ± 2	8 ± 4	8 ± 3	6 ± 1*‡
Intramucosal – arterial PCO_2 _(mmHg)	Sham	4 ± 4	5 ± 8	5 ± 8	5 ± 8	6 ± 9
	Normal	7 ± 4	6 ± 5	12 ± 5	15 ± 6*‡	19 ± 4*‡
	Increased	5 ± 7	2 ± 9	7 ± 7	12 ± 8	9 ± 6†
Systemic VCO_2 _(ml/min per kg)	Sham	5.2 ± 1.9	4.5 ± 1.2	4.0 ± 1.5	4.7 ± 1.2	4.6 ± 1.8
	Normal	6.0 ± 2.4	4.9 ± 1.4	4.9 ± 1.7	5.0 ± 1.3	5.0 ± 1.7
	Increased	6.5 ± 2.5	4.8 ± 2.4	6.1 ± 2.8	5.8 ± 2.3	5.8 ± 4.7
Intestinal VCO_2 _(ml/min per kg)	Sham	36.7 ± 10.9	38.1 ± 11.3	34.0 ± 8.8	43.2 ± 10.6	36.7 ± 5.6
	Normal	37.7 ± 10.9	35.3 ± 11.6	37.2 ± 13.7	41.8 ± 20.3	36.7 ± 16.2
	Increased	36.5 ± 21.8	35.3 ± 14.6	27.4 ± 9.4	35.8 ± 12.9	34.0 ± 7.4
Mixed venous blood capacity for transporting CO_2 _(ml/100 ml per mmHg)	Sham	0.67 ± 0.12	0.59 ± 0.40	0.51 ± 0.11	0.61 ± 0.21	0.61 ± 0.13
	Normal	0.62 ± 0.12	0.49 ± 0.12*	0.55 ± 0.04*	0.47 ± 0.09*	0.44 ± 0.09*†
	Increased	0.67 ± 0.24	0.38 ± 0.27*	0.42 ± 0.24*	0.45 ± 0.19*	0.48 ± 0.12*†
Mesenteric venous blood capacity for transporting CO_2 _(ml/100 ml per mmHg)	Sham	1.14 ± 0.24	1.15 ± 0.32	1.22 ± 0.29	1.37 ± 0.22	1.28 ± 0.08
	Normal	1.04 ± 0.22	0.99 ± 0.38	0.86 ± 0.24†	0.78 ± 0.33*†	0.76 ± 0.24*†
	Increased	1.17 ± 0.45	0.85 ± 0.29	0.66 ± 0.27†	0.81 ± 0.19†	0.69 ± 0.18*†

* *P *< 0.05 versus basal. † *P *< 0.05 versus sham. ‡ *P *< 0.05 versus normal. Sham, sham group; normal, normal blood flow group; increased, increased blood flow group.
